# Impact on Epidemic Measles of Vaccination Campaigns Triggered by Disease Outbreaks or Serosurveys: A Modeling Study

**DOI:** 10.1371/journal.pmed.1002144

**Published:** 2016-10-11

**Authors:** Justin Lessler, C. Jessica E. Metcalf, Felicity T. Cutts, Bryan T. Grenfell

**Affiliations:** 1 Department of Epidemiology, Johns Hopkins Bloomberg School of Public Health, Baltimore, Maryland, United States of America; 2 Department of Ecology and Evolutionary Biology, Princeton University, Princeton, New Jersey, United States of America; 3 Office of Population Research, Princeton University, Princeton, New Jersey, United States of America; 4 Fogarty International Center, Bethesda, Maryland, United States of America; 5 London School of Hygiene and Tropical Medicine, London, United Kingdom; Mahidol-Oxford Tropical Medicine Research Unit, THAILAND

## Abstract

**Background:**

Routine vaccination supplemented by planned campaigns occurring at 2–5 y intervals is the core of current measles control and elimination efforts. Yet, large, unexpected outbreaks still occur, even when control measures appear effective. Supplementing these activities with mass vaccination campaigns triggered when low levels of measles immunity are observed in a sample of the population (i.e., serosurveys) or incident measles cases occur may provide a way to limit the size of outbreaks.

**Methods and Findings:**

Measles incidence was simulated using stochastic age-structured epidemic models in settings conducive to high or low measles incidence, roughly reflecting demographic contexts and measles vaccination coverage of four heterogeneous countries: Nepal, Niger, Yemen, and Zambia. Uncertainty in underlying vaccination rates was modeled. Scenarios with case- or serosurvey-triggered campaigns reaching 20% of the susceptible population were compared to scenarios without triggered campaigns. The best performing of the tested case-triggered campaigns prevent an average of 28,613 (95% CI 25,722–31,505) cases over 15 y in our highest incidence setting and 599 (95% CI 464–735) cases in the lowest incidence setting. Serosurvey-triggered campaigns can prevent 89,173 (95% CI, 86,768–91,577) and 744 (612–876) cases, respectively, but are triggered yearly in high-incidence settings. Triggered campaigns reduce the highest cumulative incidence seen in simulations by up to 80%. While the scenarios considered in this strategic modeling exercise are reflective of real populations, the exact quantitative interpretation of the results is limited by the simplifications in country structure, vaccination policy, and surveillance system performance. Careful investigation into the cost-effectiveness in different contexts would be essential before moving forward with implementation.

**Conclusions:**

Serologically triggered campaigns could help prevent severe epidemics in the face of epidemiological and vaccination uncertainty. Hence, small-scale serology may serve as the basis for effective adaptive public health strategies, although, in high-incidence settings, case-triggered approaches are likely more efficient.

## Abstract 2

In a modeling study addressing measles vaccination approaches in four developing countries, Justin Lessler and colleagues investigate the relative merits of disease outbreak or serologically triggered vaccination campaigns.

## Author Summary

**Why Was This Study Done?:**

- Large, unexpected measles outbreaks continue to occur, even in settings where control measures appear effective.
- Currently, large-scale vaccination campaigns that occur at regular 2–5 y intervals are a key element of control in many settings.
- Work on outbreak response vaccination suggests that triggering campaigns in response to information about the epidemiological context of the population could help avert cases, but the appropriate design and impact of such campaigns were unclear.

**What Did the Researchers Do and Find?:**

- The researchers developed mathematical models of measles dynamics reflective of a range of demographic settings, and then simulated control strategies that included vaccination campaigns launched in response to triggers such as a threshold number of cases or the immune status of the population in a sentinel age group (as measured in a small serological survey).
- Results showed that, across a range of settings, serologically triggered campaigns could reduce incidence and prevent the most severe epidemics.
- However, in high-incidence settings, triggered campaigns may occur so often as to become essentially routine activities (in which case planned activities may be more appropriate).

**What Do These Findings Mean?:**

- Triggered campaigns might provide a powerful addition to the current landscape of public health and one of particular relevance as measles control is strengthened.
- Local context must be carefully considered when deciding on the best use of triggered campaigns.

## Introduction

Measles remains a leading cause of death among young children in low-income countries, despite considerable progress over the last decade [1]. The major measles control activities in addition to routine vaccination are supplemental immunization activities (SIAs), i.e., periodic national or regional campaigns aimed at providing vaccine to everyone in a defined age range [2]. Ultimately, the core solution to measles control lies in routine vaccination with at least two doses of measles-containing vaccine (MCV) but, in much of the world, SIAs are relied upon to achieve adequate coverage. However, even countries thought to have highly successful control sometimes experience large outbreaks because of unnoticed buildups of susceptible individuals, as recently occurred in countries such as Zambia, Malawi, and Burkina Faso [3].

The 2009 WHO guidelines for outbreak response in mortality reduction settings and the WHO Global Measles and Rubella Strategic Plan for 2012–2014 extended the classic “static” control strategies (routine immunization and SIAs occurring at intervals determined by the level of routine coverage) to include the possibility of reactive responses [2,4]. Outbreak response vaccination (ORV) was recommended for outbreaks occurring in measles mortality reduction areas [2], based on evidence that epidemics build sufficiently slowly that large numbers of cases can be averted given a sufficiently prompt reaction [5–8]. ORV has also been recommended in elimination settings, where a single case should lead to appropriate outbreak investigation and response, including vaccination of susceptible people in as wide an area as possible [9]. Identifying the most effective strategies for outbreak response immunization activities has been posed as one of the key issues in a recent review of research priorities for measles eradication [10]. However, although there has been a range of quantitative analyses on the impact of varying characteristics of static immunization strategies (e.g., the interval between SIAs) [11], investigations of the outcomes of reactive vaccination coverage are rare. Ferrari and colleagues showed that ORV might be key to reducing the case burden in the context of irregular, violent measles dynamics in Niger [12]. This inferred impact of reactive vaccination echoes theoretical analyses showing that average outbreak size grows exponentially with the delay from the start of an outbreak to the implementation of an intervention [13,14]. Beyond these few analyses, little is known.

We propose that such reactive vaccination campaigns be considered as part of a larger class of triggered campaigns (TCs). One could imagine a large number of reactive and prophylactic triggers for such campaigns—for instance, triggers based on case detection, measures of population immunity, or the recent performance of routine vaccination programs (many of these triggers were invoked for the 1994 campaign in England and Wales) [15,16]. Here we use stochastic age-structured epidemic models to compare two TC strategies with the SIA/routine vaccination combination that is currently in use.

## Methods

### Model Countries

We consider TC strategies in four hypothetical countries, representative of the demographics and WHO-United Nations Children's Fund’s (UNICEF) (WHO/UNICEF Estimates of National Immunization Coverage [WUENIC)) estimated MCV1 vaccination coverage in areas that are having varying success in measles control but have not achieved elimination. We emphasize that these scenarios are selected to capture and exemplify the essence of realistic regimes of demographics and measles control and are not intended to accurately represent any particular country, population, or measles control program.

The hypothetical countries are (A) a moderate-birth-rate, moderate-coverage country (similar to Yemen, 73% MCV1 coverage, 38 births per 1,000), (B) a high-birth-rate, moderate-coverage country (similar to Niger, 71% MCV1 coverage, 48 births per 1,000), (C) a lower-birth-rate, moderately high-coverage country (similar to Nepal, 86% MCV1 coverage, 24 births per 1,000), and (D) a high-birth-rate, moderately high-coverage country (similar to Zambia, 91% MCV1 coverage, 46 births per 1,000). Vaccination rates were roughly based on the WHO-UNICEF adjusted estimates for 2014, except for Zambia, where the approximate 2009 rate was used to illustrate a higher-coverage setting. The assumed birth rate, routine vaccine coverage, and population pyramid for each exemplar country are shown in S1 Table. The population modeled was equivalent to the size of the capital cities of the exemplar countries, to capture the size of the population where the random mixing assumptions of the epidemic models used most nearly hold and to avoid the need to account for geographic population structure in our results.

### Intervention Scenarios

In each country, we assumed that routine coverage was constant at the hypothetical country baseline or varied around that baseline as a random walk (see below). In all simulations, regularly planned SIAs with 80% coverage of the target population of children between 9 and 59 mo of age were modeled to occur every 5 y. Five years was selected as the maximum interval between SIAs that would give each birth cohort (with the exception of those born in the 9 mo prior to the SIA) two vaccination opportunities from the combination of routine vaccination and regularly scheduled SIAs. As with the hypothetical countries, these scenarios are meant to capture a not-implausible baseline intervention scenario that allows for comparison of TC strategies while minimizing the number of free (i.e., changeable) variables in the model (WHO recommendations are for SIAs every 2–4 y depending on routine vaccine program performance).

We compared this default control program with TCs triggered by the occurrence of N or more cases in a bi-week (when <N occurred in the previous bi-week) or the measurement of less than 85% measles seroprevalence in a sampled sentinel population. Based on informal discussions with colleagues at organizations working in measles control (e.g., WHO and Médecins Sans Frontières), case-based triggers of N = 10 and N = 25 were considered. Two sentinel populations were considered for seroprevalence studies: children from 24–36 mo of age and those from 2–5 y of age. Each August (the low measles transmission season in our simulations), serological measurements were performed in a representative random sample of 200 individuals from each sentinel population. Once a TC is triggered, there is a delay of 3 mo from the date of the survey to allow for reporting and logistical delays (longer and shorter delays are considered in a sensitivity analysis), and then a campaign is conducted that is assumed to reach 20% of the hypothetical country’s unimmunized population who are 6–59 mo of age (i.e., though a much higher percentage of the overall target population may receive MCV in the TC, only 20% of those who have not been previously successfully vaccinated will do so). The low effective coverage was selected to reflect the fact that such “just in time” campaigns are likely to be less effective at reaching previously unvaccinated populations than typical SIAs, as well as the difficulty in reaching chronically unvaccinated populations (alternate coverages were examined in sensitivity analyses) [17]. In subanalyses, we also consider the effect of targeting a wide age range (6 mo olds to 15 y olds), motivated by the wide age range of measles cases seen in some outbreaks after a long period of successful control [18]. The measles vaccine is assumed to function as an “all or nothing” vaccine (i.e., it induces full or no immunity), with vaccine effectiveness increasing with age during the first year of life as specified in Lessler et al. [19]. In our models, those without immunity after receiving a dose of vaccine have the same opportunity to be vaccinated and protected in other rounds as if they had not previously received a dose of vaccine. Routine vaccination is considered to reach the assumed level at 24 mo of age, with the age distribution of coverage following the same pattern as was measured for Zambia in previous work [19].

In further simulations, to capture realistic deviations from presumed routine coverage, we implemented an autocorrelated random walk, designed to reflect a chosen variance, τ^2^, over 10 y. At every time-step, we generated a random deviate from a normal distribution with mean 0 and variance set to τ^2^ / [24 x 10]. If the probability density of this candidate value based on a normal distribution with mean 0 and variance τ^2^ was greater than a random deviate from a uniform distribution, the candidate was adopted; if not, the previous value was retained. The true value of routine coverage at each time-step was obtained by increasing or reducing current vaccination rates by this value. To capture realistic deviations from presumed SIA coverage, we generated random deviates from a normal distribution with mean 0 and variance identical to that used for routine vaccination; this was added to the presumed level of SIA coverage.

In addition to the default scenario described above, we considered alternate coverage levels for TCs (10% and 40% of susceptibles covered), differing delays between a TC being triggered and implemented (1 mo and 6 mo) and an alternate level for the case-based trigger (ten cases).

### Simulations and Comparison

We performed stochastic simulations (200 for constant-routine vaccination-rate scenarios, 1,500 for varying-routine vaccination-rate scenarios) of each intervention scenario for each country using a seasonally forced, age-structured time-series susceptible infected recovered (TSIR) model, details of which can be found in Metcalf et al. (2012) [20]. Each simulation was run for 20 y after the introduction of a single measles case in a fully susceptible population with only routine vaccination and SIAs used for control. After these 20 y of “burn in,” additional intervention strategies were introduced, and the simulation was run for an additional 15 y. Intervention scenarios were compared based upon the total number of cases, the number of cases averted, and the frequency at which TCs were triggered.

Additional methodological details are provided in S1 Text.

## Results

**Fig 1:**
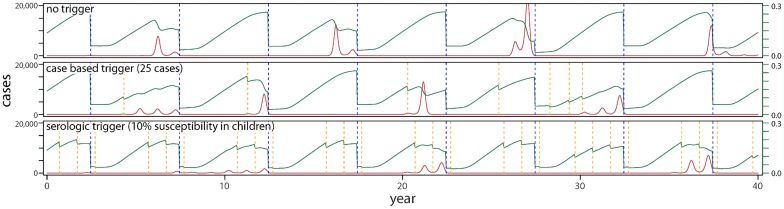
Typical time-series of incidence for three vaccination scenarios in a Yemen-like population: (i) a baseline scenario of routine vaccination combined with SIAs every 5 y; (ii) this baseline combined with campaigns that are triggered by a threshold number of cases; and (iii) this baseline combined with campaigns triggered by a threshold degree of serology in a target population age range. Red lines indicate number of cases, green lines susceptibility in children under 5 y of age, dashed blue lines the time of SIA occurrence, and dashed orange lines the timing of TCs.

**Fig 2:**
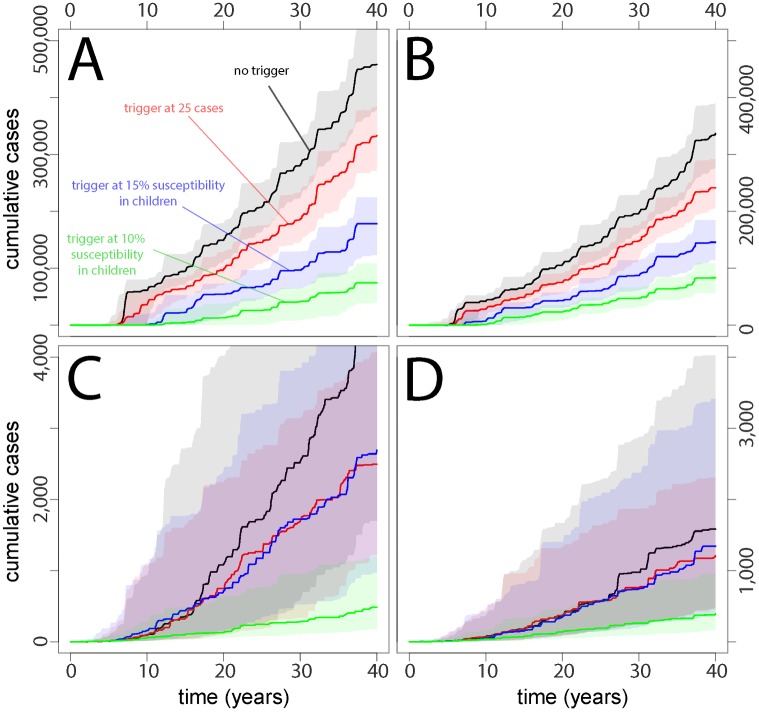
Cumulative case burdens in (A) Yemen-like, (B) Niger-like, (C) Nepal-like, and (D) Zambia-like populations for a range of different types of vaccination scenarios, where TCs are delayed by 3 mo relative to the trigger and TC vaccination reaches 20% of the susceptible population.

**Fig 3:**
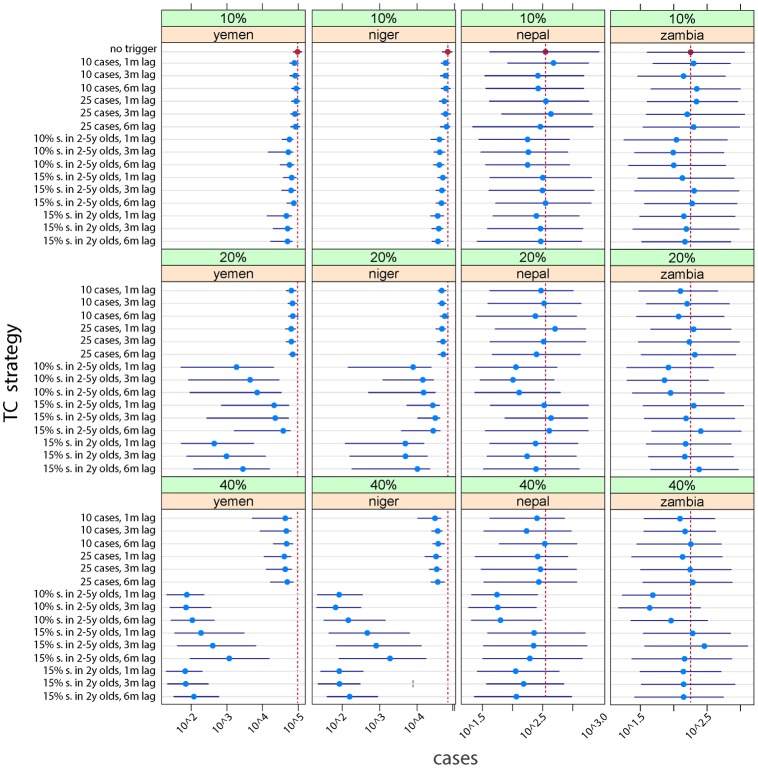
Median (point) and interquartile range (lines) of the cumulative case burden after 15 y for 200 simulations across a range of different vaccination scenarios (no trigger, case-based trigger, and serological-based triggers) with different delays before the campaign can be deployed (1 mo, 3 mo, and 6 mo), different coverage attained in the TCs (10%, 20%, or 40%), different degrees of sensitivity in the serological surveys (10% or 15%), and different age ranges for the serological surveys (children, aged 1 to 5 y; or infants, aged 24 to 36 mo). The baseline scenario (no trigger) is shown as a vertical red line for each country.

For each country, we simulated time-series of measles incidence and critical vaccination events for both the baseline scenario (no TCs, routine vaccination based on the country setting, and SIAs every 5 y) and the baseline scenario plus TCs (case-based or serologic) (Fig 1). The projected cumulative measles incidence in each scenario shows clear benefits from TCs compared to static vaccination strategies (Fig 2). In Yemen- and Niger-like settings, where routine coverage is relatively low, serologically triggered campaigns prevent more cases than both case-based triggers and no-TC scenarios (Figs 2 and 3). In Nepal- and Zambia-like countries, where routine coverage is relatively high, TCs are generally better than the baseline case (no triggers), but the benefits are less stark and are dwarfed by expected variation from stochastic variability. In these situations, the expected benefits of all but the most sensitive serologically triggered campaigns compared to case base methods are less clear.

Fig 3 shows the effects on cumulative case burden after 15 y of an array of different strategies with different degrees of coverage and delays before TC implementation. Relative to the baseline scenario (no triggers, vertical red line) the effect of TCs is considerably more marked in contexts where baseline coverage is lower (e.g., Yemen-like countries), especially where a reasonable degree of coverage can be attained in the TCs. However, an effect can be seen in all scenarios. In some contexts, monitoring only 2 y olds (24–36 mo of age) for higher levels of susceptibility (15%) achieves similar outcomes to monitoring children aged 2–5 y with a more sensitive cutoff (10%) and may be logistically easier.

The greater impact of TCs in low-coverage situations is largely the result of more frequent TCs. In the Yemen-like situation, a sensitive serologic trigger (i.e., 10% susceptibility in children) leads to TCs being conducted in 10 out of the first 15 y from policy implementation, essentially becoming a routine event (Table 1, S1, S2 and S3 Tables). While case-triggered approaches lead to a smaller reduction in cumulative incidence, they occur far less frequently and tend to result in more cases prevented per campaign in lower-coverage (Yemen-like) situations. In higher-coverage situations, serologically triggered TCs occur far less often, and using a sensitive susceptibility trigger of 10% appears to be able to greatly reduce the size of epidemics. In these settings, case-based, or higher (15%) susceptibility, triggers have less impact.

**Table 1:** Mean total number of cases averted after 15 y across 1,500 simulations, with noisy vaccination trajectories and mean total number of TCs and cases averted per TC for vaccination scenarios with the stated trigger, 20% coverage, and a 3-mo lag between trigger and vaccination.

	Cases Averted (95% CI)	Average Number of TCs (95% CI)	Cases Averted per TC (95% CI)
**Yemen-like** (73% routine coverage, 38 births per 1,000)			
*10 cases*	27,705 (24,780–30,631)	2.18 (2.10–2.25)	12,736 (11,322–14,150)
*25 cases*	28,613 (25,722–31,505)	2.09 (2.02–2.16)	13,691 (12,223–15,158)
*10% s in 2–5 y olds*	83,722 (81,247–86,198)	9.81 (9.77–9.85)	8,537 (8,282–8,792)
*15% s in 2–5 y olds*	68,084 (65,336–70,832)	7.18 (7.13–7.24)	9,480 (9,090–9,869)
*15% s in 2 y olds*	89,173 (86,768–91,577)	11.01 (10.97–11.06)	8,098 (7,877–8,319)
**Niger-like** (71% routine coverage, 48 births per 1,000)			
*10 cases*	18,269 (16,363–20,175)	2.12 (2.06–2.19)	8,612 (7,675–9,549)
*25 cases*	18,422 (16,479–20,364)	1.95 (1.88–2.01)	9,470 (8,423–10,516)
*10% s in 2–5 y olds*	52,718 (51,022–54,414)	10.15 (10.11–10.20)	5,193 (5,024–5,362)
*15% s in 2–5 y olds*	41,124 (39,280–42,968)	7.53 (7.47–7.58)	5,463 (5,214–5,711)
*15% s in 2 y olds*	56,207 (54,570–57,844)	11.45 (11.41–11.48)	4,910 (4,766–5,054)
**Nepal-like** (86% routine coverage, 24 births per 1,000)			
*10 cases*	712 (571–852)	1.39 (1.32–1.46)	513 (408–617)
*25 cases*	582 (436–728)	0.91 (0.85–0.96)	640 (475–806)
*10% s in 2–5 y olds*	1,093 (960–1226)	5.03 (4.98–5.09)	217 (191–244)
*15% s in 2–5 y olds*	416 (258–574)	1.20 (1.16–1.25)	346 (214, 478)
*15% s in 2 y olds*	701 (554–848)	2.18 (2.12–2.24)	322 (254, 389)
**Zambia-like** (91% routine coverage, 46 births per 1,000)			
*10 cases*	599 (464–735)	1.07 (1.01–1.13)	561 (430–692)
*25 cases*	459 (320–598)	0.74 (0.69–0.79)	621 (429–813)
*10% s in 2–5 y olds*	744 (612–876)	3.72 (3.67–3.77)	200 (164–236)
*15% s in 2–5 y olds*	108 (−70 to 285)	0.38 (0.35–0.41)	284 (−185 to 752)
*15% s in 2 y olds*	326 (174–479)	0.88 (0.83–0.92)	373 (197–549)

Abbreviations: s, seropositive

**Fig 4:**
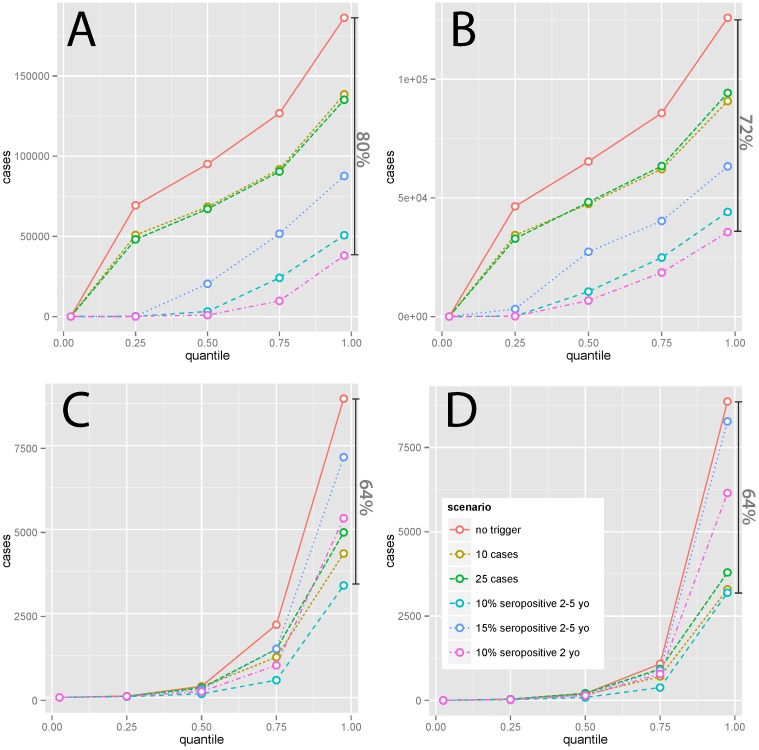
Percentiles (2.5, 25, 50, 75, and 97.5) of simulated (1,500 simulations, noisy vaccination rate) 15-y cumulative measles incidence under different intervention scenarios with 20% additional coverage in (A) Yemen-like, (B) Niger-like, (C) Nepal-like, and (D) Zambia-like populations. Dark vertical bars indicate the percent reduction in the largest (97.5th percentile) epidemics seen achieved by the most effective intervention considered.

The expected (i.e., average) effect of a reactive intervention on case burden is clearly of interest; however, it may not be the most important public health outcome. Instead, we may be more concerned with preventing less common, but disastrous, large epidemics. While simulations suggest a strong benefit of TCs in reducing the median simulated cumulative incidence only in high-incidence (i.e., Yemen- and Niger-like) settings, TCs limit the size of the largest epidemics seen in all settings. In simulations with stochasticity in underlying vaccination rates, the most effective serology-based TCs reduced the size of the largest epidemics seen (i.e., the 97.5th percentile of size) by 64% or more in all scenarios (Fig 4, S4 Fig). Hence, TCs may be effective in preventing the worst epidemics in all settings. In low-incidence settings, case-based approaches can be nearly as effective for preventing extremely large outbreaks as serological approaches (Fig 4), although serological approaches are more effective overall.

Subanalyses in which TCs target 6 mo olds to 15 y olds show qualitatively similar results to the main analysis (S2, S3, and S4 Figs, and S4 Table). Improvements are significant, for case-trigger-based TCs in high-incidence settings, in some instances preventing over 150% as many cases, but tend to be less pronounced for serologically triggered TCs and in low-incidence settings, where in most instances differences are not significant.

## Discussion

This analysis explored the impact of TCs across a range of demographic and measles vaccine coverage contexts. We found that TCs consistently reduced the cumulative case burden. Furthermore, in all settings, TCs were effective in preventing rare yet large and potentially most damaging measles epidemics. We found that this positive impact is observed even for campaigns reaching 20% or less of the unimmunized population, and the largest impact is observed for serologically triggered surveys. Overall, however, the magnitude of the impact of TCs is highly dependent on the demographic and vaccine coverage context considered. Some TC strategies may be triggered so often that they become highly inefficient (i.e., few cases are prevented per campaign). Consequently, our results suggest that although TCs could powerfully strengthen measles control programs in some settings, they are not appropriate to all settings.

Where routine coverage is relatively high and measles well controlled, as is the case in the Nepal- and Zambia-like scenarios, case-based triggers are not very effective in terms of cases averted per campaign (S1 Table). This is because when outbreaks occur, they will tend to be of short duration, given the rarity of susceptibles, so that campaigns triggered by the start of any outbreak will only avert a small number of cases. Serology-based triggers (essentially triggered prophylactic vaccination) can help prevent outbreaks before they begin and thus prevent more cases per campaign. This underlines the major potential benefits of susceptibility monitoring as an effective form of risk management by serology, when logistically feasible [21]. However, in low-incidence settings, both serology- and case-based approaches will rarely be triggered (in many simulations for Zambia- and Nepal-like settings, no TC was triggered), but having a TC program in place effectively avoids the worst outcomes in terms of large measles epidemics and can provide a safeguard against the effects of overestimation of vaccination coverage in routine programs or SIAs (Fig 4).

If measles is less well controlled, as in our Yemen- and Niger-like scenarios, serology-based triggers would lead to a TC almost every year (Fig 1, Table 1, and S1 Table). This suggests that rather than the additional investment in a serological survey every year, simply supplementing routine vaccination by a yearly campaign might be more effective, if the required investment in campaigns is considered acceptable. By contrast, case-triggered campaigns occur much less frequently and are effective (though less so) in averting cases, as outbreaks tend to be of long duration. Case-based TCs should consequently be the supplementary strategy of choice if resource constraints mean that scheduled campaigns are not implemented often enough and that routine coverage is not increased.

Just as serology-based campaigns will be inefficient in contexts where measles is poorly controlled, more broadly, any TC strategy will not be suitable for countries with particularly low measles vaccination coverage and large yearly epidemics. In these settings, any TC trigger will likely be activated every year, and the approach will cease to be one of TCs and will become one of regular yearly supplemental vaccination activities covering a broad age range. While such activities would undoubtedly aid in measles control, they would merely be a poorly planned version of a strategy of yearly SIAs.

Serosurveys covering wider age ranges than considered here may capture some important gaps in immunity in older age groups and better reflect relevant population susceptibility, but may yield limited benefits over an ongoing program of regular serosurveys of a narrower age group in which each cohort would have been surveyed at an earlier age. Targeting a small, young age group will also increase power if the size of the serosurvey is limited, as an equal-sized survey spread across larger age groups will have more noise and be less likely to detect cohorts of high susceptibility. This higher sensitivity is one reason for the superior performance of triggers focused on 24–36 mo olds in our simulations. However, if only young individuals are tested, trends in serosurvey results year-to-year should be carefully tracked.

TCs remain a sparsely deployed public health strategy. Case-triggered campaigns are the most common, particularly in situations in which elimination has been achieved (e.g., the United States) [22]. For nonelimination situations, in low- and middle-income countries, a recent review found that out of 461 outbreaks reported in the literature, only 38 referred to case-triggered campaigns [7]. We could not identify any clear serologically triggered campaigns (although serology contributed to the 1994 campaign in the United Kingdom [15,16] and led to continued use of polio vaccine in US army recruits [DS Burke, personal communication] [23]). Our results indicate that this might be a fruitful area for further development of public health strategy. Having a TC strategy in place might be particularly useful in countries with high (>90%) purported vaccination coverage, serving as a safeguard against accumulations of immunity if routine SIAs are no longer considered to be needed.

The costs of a TC program can be divided into three categories: (1) the cost of ongoing monitoring, (2) the costs of conducting each campaign, and (3) the cost of having resources available to implement an emergency campaign. If surveillance programs are already robust, case-based TC programs would incur minimal excess costs, if any; however, in countries where surveillance is poor, small, highly targeted serosurveys may actually be cheaper than system-wide improvements in case detection (although the latter is needed in any case if countries are to move towards elimination). The bulk of additional costs will come from campaigns themselves and include direct costs such as vaccine doses, personnel, and equipment, as well as indirect costs incurred from diverting resources from other health activities. These costs are proportional to the number of campaigns performed; hence, they will be greatest when serosurvey-based triggers are used in high-incidence settings. The costs of having resources at the ready are potentially significant, but costs could be minimized by having a shared stockpile that countries could draw from in an emergency (e.g., the International Coordinating Group oral cholera vaccine stockpile).

Strategic modeling studies such as this require a balance of realism with abstraction and generalization. Hence, there are many limitations in our study that affect the quantitative interpretation of this analysis in relation to any particular country or setting. Countries were assumed to have demographic, but not spatial, structure, and such spatial heterogeneities can have a considerable effect on dynamics and campaign performance if certain areas are systematically missed. We used a highly simplified model of measles vaccination policy, and current practice of basing the frequency and age-range of SIAs on routine vaccination program performance and measles incidence may achieve some of the reductions in incidence achieved by TCs. Likewise, while the control strategies here are illustrative of those that might be considered, they ignore some complexities (e.g., the proportion of cases detected by surveillance) and are not optimized for each setting. Before implementing a TC-based strategy, countries may want to perform additional analyses based on their measles control goals and adjustfor the performance of their surveillance system. Even in simulation studies, it is not practical to create a perfect counterfactual for every instance of an epidemic and control strategy; hence, we can only compare strategies using aggregate statistics. Further, there is currently very little data on the costs of implementation of the various components of a triggered vaccination strategy, and further research is needed to evaluate the investment case for TCs. Despite these limitations, our study provides insights into the potential effectiveness of triggered control strategies and the context in which such strategies might or might not be effective.

While our focus here was on measles in countries with stable vaccination programs, TC campaigns may be relevant for other vaccines within the Expanded Program on Immunization. One key advantage of expanding the program might be that serological surveys could be combined, maximizing returns on that investment, which our analysis also shows can be effective even for relatively modest-sized surveys (e.g., 200 people in cities of 1–2 million). Likewise, TCs may also play an important role in the pathway to elimination when routine vaccine coverage is increasing, serving as a potential safeguard as SIAs are being phased out, and the optimal use of TCs in such situations is an important area for future research. However, an important caveat is that all TC strategies may be vulnerable to underlying heterogeneities in case numbers or susceptibility profiles (for example, spatial heterogeneities within cities or across countries), which may result in either too frequent or too rare triggering. The potential for greater use of serological monitoring of population susceptibility to vaccine-preventable infections and its use both for triggering supplementary control activities and for monitoring progress towards elimination targets needs further investigation.

## Supporting Information

S1 Fig Percentiles (2.5, 25, 50, 75, and 97.5) of simulated (200 simulations, constant vaccination rate) 15-y cumulative measles incidence under different intervention scenarios in (A) Yemen-like, (B) Niger-like, (C) Nepal-like, and (D) Zambia-like populations. Dark vertical bars indicate the percent reduction in the largest (97.5th percentile) epidemics seen achieved by the most effective intervention considered.
(TIF)

S2 Fig Cumulative case burdens when TCs target 6 mo olds to 15 y olds in (A) Yemen-like, (B) Niger-like, (C) Nepal-like, and (D) Zambia-like populations for a range of different types of vaccination scenarios, where TCs are delayed by 3 mo relative to the trigger, and TC vaccination reaches 20% of the unvaccinated population. (TIF)

S3 Fig When TCs target 6 mo olds to 15 y olds, median (point) and interquartile range (lines) of the cumulative case burden after 15 y for 200 simulations across a range of different vaccination scenarios (no trigger, case-based trigger, and serological-based triggers) with different delays before the campaign can be deployed (1 mo, 3 mo, and 6 mo), different coverage attained in the TCs (10%, 20%, or 40%), different degrees of sensitivity in the serological surveys (10% or 15%), and different age ranges for the serological surveys (children, aged 1 to 25 y; or infants, aged 24 to 36 mo). The baseline scenario (no trigger) is shown as a vertical red line for each country.
(TIF)

S4 Fig Percentiles (2.5, 25, 50, 75, 97.5) of simulated (200 simulations, constant vaccination rate) 15-y cumulative measles incidence under different intervention scenarios where TCs target 6 mo olds to 15 y olds in (A) Yemen-like, (B) Niger-like, (C) Nepal-like, and (D) Zambia-like populations. Dark vertical bars indicate the percent reduction in the largest (97.5th percentile) epidemics seen achieved by the most effective intervention considered.
(TIF)

S1 Table Birthrates, vaccination rates, and population pyramids for four scenarios. (PDF)

S2 Table Mean number of cases averted after 15 y across 200 simulations, median total number of TCs for the full range of vaccination scenarios, and their ratio, i.e., number of cases averted per campaign. (DOCX)

S3 Table Mean number of cases averted after 15 y across 1,500 simulations with noisy vaccination trajectories, median total number of TCs for the full range of vaccination scenarios, and their ratio, i.e., number of cases averted per campaign. (DOCX)

S4 Table Performance when TCs target 6 mo olds to 15 y olds. Columns are as follows: mean total number of cases averted after 15 y across 200 simulations, mean total number of TCs and cases averted per TC for vaccination scenarios with the stated trigger, 20% coverage, and a 3-molag between trigger and vaccination.
(DOCX)

S1 Text Supplementary methods and details on model structure. (DOCX)

## Abbreviations

MCV: measles-containing vaccine
ORV: outbreak response vaccination
SIA: supplemental immunization activity
TC: triggered campaign
TSIR: time-series susceptible infected recovered
UNICEF: The United Nations Children's Fund
WUENIC: WHO/UNICEF Estimates of National Immunization Coverage
