# Supplementary figures and images for: Impact on Epidemic Measles of Vaccination Campaigns Triggered by Disease Outbreaks or Serosurveys: A Modeling Study
Source: PLoS Med. 2016 Oct 11;13(10):e1002144. doi: 10.1371/journal.pmed.1002144 (PMC5058560; doi:10.1371/journal.pmed.1002144)

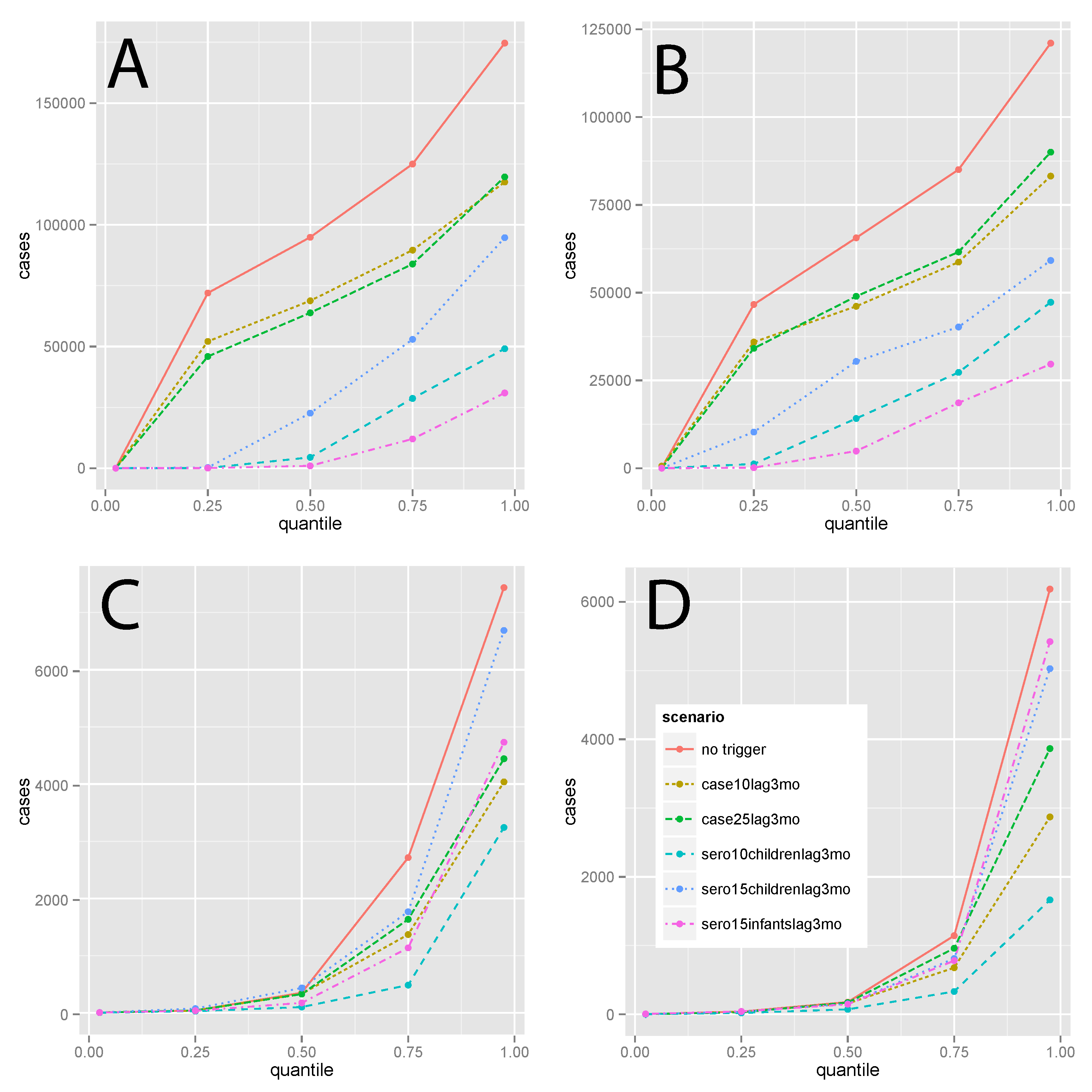

Supplement: S1 Fig — Dark vertical bars indicate the percent reduction in the largest (97.5th percentile) epidemics seen achieved by the most effective intervention considered. (TIF) [file pmed.1002144.s001.tif]

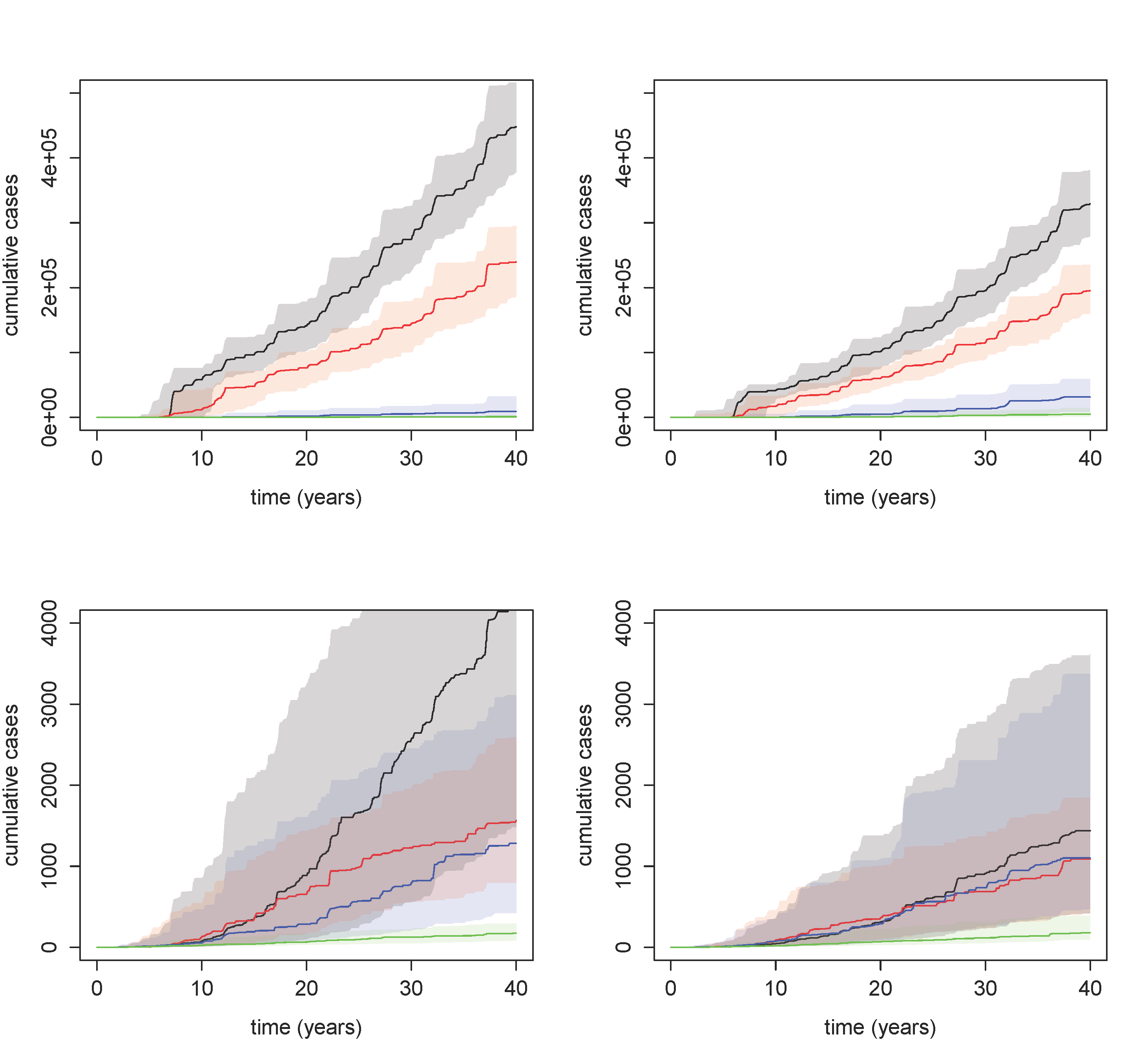

Supplement: S2 Fig — (TIF) [file pmed.1002144.s002.tif]

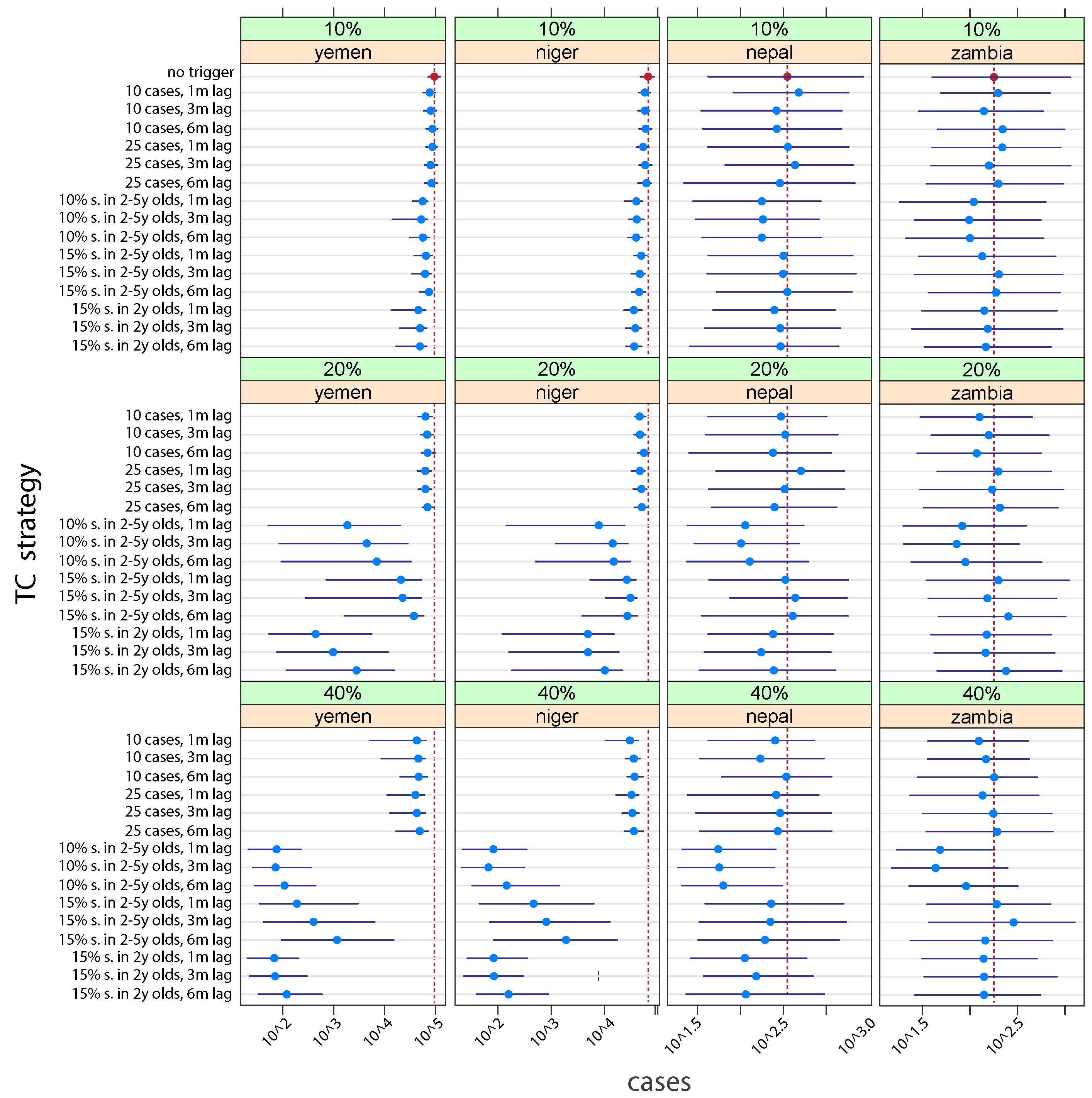

Supplement: S3 Fig — The baseline scenario (no trigger) is shown as a vertical red line for each country. (TIF) [file pmed.1002144.s003.tif]

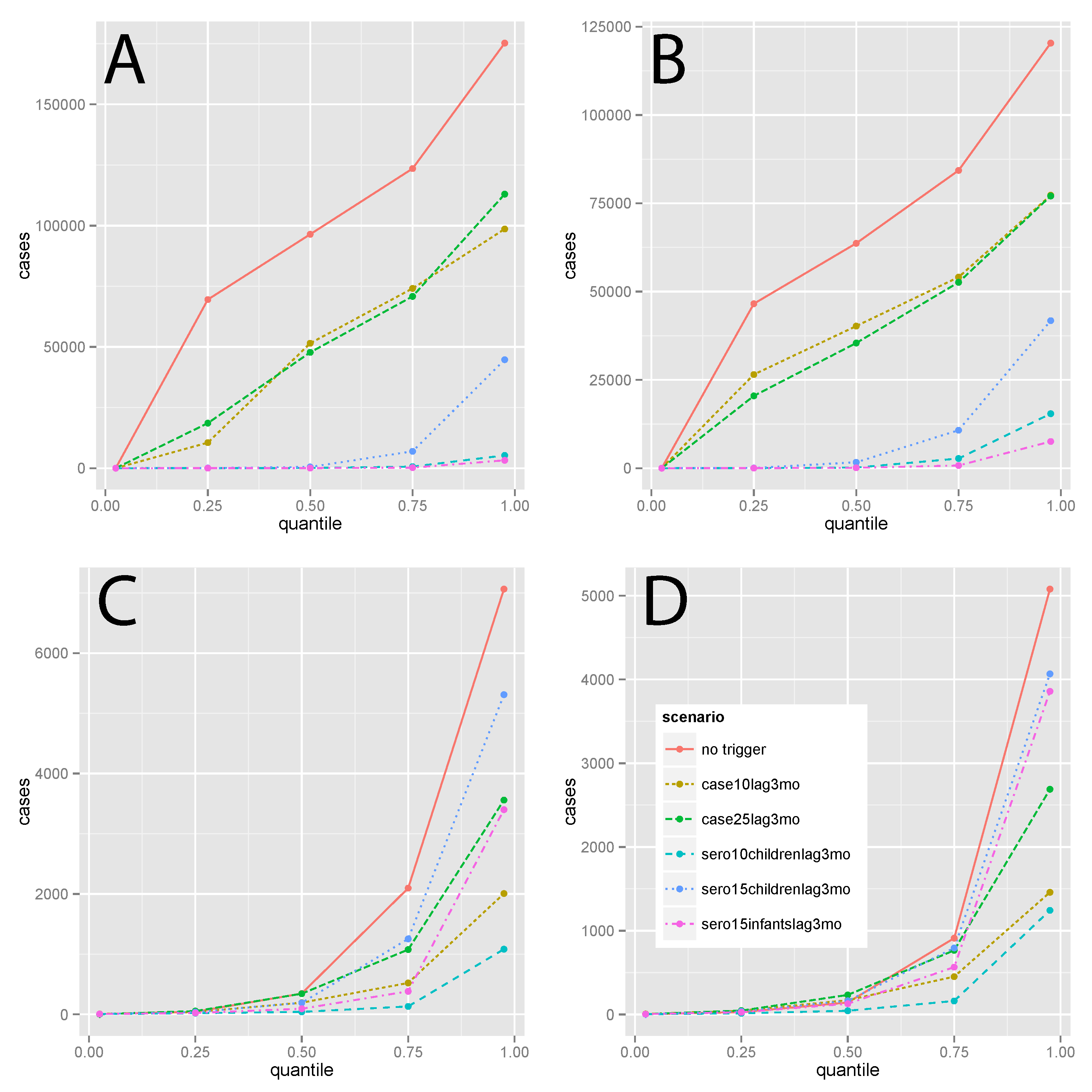

Supplement: S4 Fig — Dark vertical bars indicate the percent reduction in the largest (97.5th percentile) epidemics seen achieved by the most effective intervention considered. (TIF) [file pmed.1002144.s004.tif]
