# Supplementary material for: Impact on Epidemic Measles of Vaccination Campaigns Triggered by Disease Outbreaks or Serosurveys: A Modeling Study
Source: PLoS Med. 2016 Oct 11;13(10):e1002144. doi: 10.1371/journal.pmed.1002144 (PMC5058560; doi:10.1371/journal.pmed.1002144)
Supplement: S1 Table — (PDF) [file pmed.1002144.s005.pdf]

Country	Birth Rate (per 1000)	Routine Vaccination Rate	Pop Pyramid (1-90 years)
**Yemen-like**	38	73%	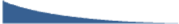
**Niger-like**	48	71%	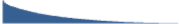
**Nepal-like**	24	86%	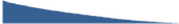
**Zambia-like**	46	91%	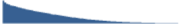
