# Supplementary material for: Impact on Epidemic Measles of Vaccination Campaigns Triggered by Disease Outbreaks or Serosurveys: A Modeling Study
Source: PLoS Med. 2016 Oct 11;13(10):e1002144. doi: 10.1371/journal.pmed.1002144 (PMC5058560; doi:10.1371/journal.pmed.1002144)
Supplement: S2 Table — (DOCX) [file pmed.1002144.s006.docx]

|  | **Yemen-like** | | | **Niger-like** | | | **Nepal-like** | | | **Zambia-like** | | |
| --- | --- | --- | --- | --- | --- | --- | --- | --- | --- | --- | --- | --- |
|  | **cases**  **averted** | **TCs** | **c. averted/**  **TC** | **cases**  **averted** | **TCs** | **c. averted/**  **TC** | **cases**  **averted** | **TCs** | **c. averted/**  **TC** | **cases**  **averted** | **TCs** | **c. averted/**  **TC** |
| **10% of unvaccinated covered** | | | | | | | | | | | | |
| 10 cases, 1m lag | 19722.7 | 2 | 9861.3 | 8961.5 | 2 | 4480.8 | 344.9 | 1 | 344.9 | 334.1 | 1 | 334.1 |
| 10 cases, 3m lag | 19484.3 | 2 | 9742.1 | 10541.6 | 2 | 5270.8 | 528.7 | 1 | 528.7 | 274.2 | 1 | 274.2 |
| 10 cases, 6m lag | 11631.1 | 2 | 5815.6 | 7997.7 | 2 | 3998.9 | 592.6 | 1 | 592.6 | 237.4 | 1 | 237.4 |
| 25 cases, 1m lag | 12354.8 | 2 | 6177.4 | 11803.9 | 2 | 5901.9 | 367.6 | 1 | 367.6 | 209.8 | 0 | – |
| 25 cases, 3m lag | 14043.6 | 1 | 14043.6 | 8753.6 | 2 | 4376.8 | 380.8 | 1 | 380.8 | 182.5 | 0 | – |
| 25 cases, 6m lag | 13483.5 | 2 | 6741.7 | 7446.6 | 2 | 3723.3 | 304.0 | 0 | -- | 180.2 | 0 | – |
| 10% s in 2-5y olds, 1m lag | 45237.8 | 10 | 4523.8 | 29988.5 | 10 | 2998.9 | 826.2 | 6 | 137.7 | 244.8 | 4 | 61.2 |
| 10% s in 2-5y olds, 3m lag | 49684.5 | 10 | 4968.4 | 27495.3 | 10 | 2749.5 | 901.6 | 6 | 150.3 | 372.3 | 4 | 93.1 |
| 10% s in 2-5y olds, 6m lag | 43350.1 | 10 | 4335.0 | 29045.6 | 10 | 2904.6 | 864.1 | 6 | 144.0 | 415.2 | 4 | 103.8 |
| 15% s in 2-5y olds, 1m lag | 34609.1 | 8 | 4326.1 | 20935.3 | 8 | 2616.9 | 312.1 | 1 | 312.1 | 156.6 | 0 | – |
| 15% s in 2-5y olds, 3m lag | 38381.5 | 8 | 4797.7 | 22830.8 | 8 | 2853.9 | 194.1 | 1 | 194.1 | 52.1 | 0 | – |
| 15% s in 2-5y olds, 6m lag | 31329.8 | 7 | 4475.7 | 22959.7 | 8 | 2870.0 | 297.2 | 1 | 297.2 | -60.5 | 0 | – |
| 15% s in 2y olds, 1m lag | 52721.7 | 12 | 4393.5 | 32118.6 | 12 | 2676.6 | 680.9 | 2 | 340.5 | 268.1 | 1 | 268.1 |
| 15% s in 2y olds, 3m lag | 51151.8 | 12 | 4262.7 | 31558.5 | 11 | 2869.0 | 501.6 | 2 | 250.8 | 20.2 | 1 | 20.2 |
| 15% s in 2y olds, 6m lag | 50990.3 | 11 | 4635.5 | 31284.7 | 11 | 2844.1 | 607.2 | 2 | 303.6 | 90.8 | 1 | 90.8 |
| 20% of unvaccinated covered | | | | | | | | | | | | |
| 10 cases, 1m lag | 32624.1 | 2 | 16312.0 | 20518.5 | 2 | 10259.3 | 841.7 | 1 | 841.7 | 612.2 | 1 | 612.2 |
| 10 cases, 3m lag | 30872.2 | 2 | 15436.1 | 19456.7 | 2 | 9728.3 | 689.7 | 1 | 689.7 | 493.4 | 1 | 493.4 |
| 10 cases, 6m lag | 25044.7 | 2 | 12522.4 | 12304.5 | 2 | 6152.2 | 753.4 | 1 | 753.4 | 469.6 | 1 | 469.6 |
| 25 cases, 1m lag | 33584.8 | 2 | 16792.4 | 22410.9 | 2 | 11205.5 | 596.0 | 1 | 596.0 | 401.1 | 0 | – |
| 25 cases, 3m lag | 33260.9 | 2 | 16630.4 | 19071.4 | 2 | 9535.7 | 620.6 | 1 | 620.6 | 291.7 | 0 | – |
| 25 cases, 6m lag | 25060.7 | 1.5 | 16707.2 | 16135.7 | 2 | 8067.8 | 680.7 | 1 | 680.7 | 347.4 | 0.5 | 694.8 |
| 10% s in 2-5y olds, 1m lag | 84842.9 | 10 | 8484.3 | 54058.4 | 10 | 5405.8 | 1165.4 | 5 | 233.1 | 555.9 | 4 | 139.0 |
| 10% s in 2-5y olds, 3m lag | 82250.7 | 10 | 8225.1 | 50589.9 | 10 | 5059.0 | 1115.0 | 5 | 223.0 | 703.4 | 4 | 175.8 |
| 10% s in 2-5y olds, 6m lag | 80046.6 | 9 | 8894.1 | 49901.0 | 10 | 4990.1 | 1097.5 | 5 | 219.5 | 466.4 | 4 | 116.6 |
| 15% s in 2-5y olds, 1m lag | 66529.3 | 7 | 9504.2 | 42476.1 | 8 | 5309.5 | 339.1 | 1 | 339.1 | -74.1 | 0 | – |
| 15% s in 2-5y olds, 3m lag | 66681.3 | 7 | 9525.9 | 39900.7 | 8 | 4987.6 | 372.4 | 1 | 372.4 | 144.0 | 0 | – |
| 15% s in 2-5y olds, 6m lag | 60273.3 | 7 | 8610.5 | 41564.4 | 7 | 5937.8 | 317.2 | 1 | 317.2 | 82.4 | 0 | – |
| 15% s in 2y olds, 1m lag | 91256.7 | 12 | 7604.7 | 58331.6 | 12 | 4861.0 | 746.0 | 2 | 373.0 | 260.7 | 1 | 260.7 |
| 15% s in 2y olds, 3m lag | 89144.8 | 11 | 8104.1 | 57322.9 | 12 | 4776.9 | 678.1 | 2 | 339.1 | 162.4 | 1 | 162.4 |
| 15% s in 2y olds, 6m lag | 86343.6 | 11 | 7849.4 | 54467.3 | 11 | 4951.6 | 645.4 | 2 | 322.7 | 122.1 | 1 | 122.1 |
| **40% of unvaccinated covered** | | | | | | | | | | | | |
| 10 cases, 1m lag | 55858.9 | 2 | 27929.5 | 37947.4 | 2 | 18973.7 | 1048.8 | 1 | 1048.8 | 732.1 | 1 | 732.1 |
| 10 cases, 3m lag | 53825.8 | 2 | 26912.9 | 32856.8 | 2 | 16428.4 | 952.1 | 1 | 952.1 | 635.6 | 1 | 635.6 |
| 10 cases, 6m lag | 47995.6 | 2 | 23997.8 | 27967.2 | 2 | 13983.6 | 818.9 | 1 | 818.9 | 534.5 | 1 | 534.5 |
| 25 cases, 1m lag | 55637.0 | 2 | 27818.5 | 36880.5 | 2 | 18440.2 | 987.2 | 1 | 987.2 | 594.0 | 0 | – |
| 25 cases, 3m lag | 54729.9 | 2 | 27364.9 | 34610.5 | 2 | 17305.3 | 834.6 | 1 | 834.6 | 434.8 | 0 | – |
| 25 cases, 6m lag | 47269.2 | 1 | 47269.2 | 28037.2 | 2 | 14018.6 | 794.2 | 0 | – | 369.9 | 0 | – |
| 10% s in 2-5y olds, 1m lag | 95794.2 | 8 | 11974.3 | 66352.9 | 9 | 7372.5 | 1330.0 | 4 | 332.5 | 800.9 | 3 | 267.0 |
| 10% s in 2-5y olds, 3m lag | 95674.1 | 8 | 11959.3 | 66505.4 | 9 | 7389.5 | 1318.5 | 4 | 329.6 | 728.9 | 3 | 243.0 |
| 10% s in 2-5y olds, 6m lag | 95498.6 | 8 | 11937.3 | 65621.4 | 8 | 8202.7 | 1302.8 | 3 | 434.3 | 597.2 | 3 | 199.1 |
| 15% s in 2-5y olds, 1m lag | 91404.8 | 5 | 18281.0 | 62103.9 | 6 | 10350.6 | 544.3 | 1 | 544.3 | 180.6 | 0 | – |
| 15% s in 2-5y olds, 3m lag | 90127.7 | 5 | 18025.5 | 59926.0 | 6 | 9987.7 | 521.7 | 1 | 521.7 | -15.7 | 0 | – |
| 15% s in 2-5y olds, 6m lag | 84373.7 | 5 | 16874.7 | 57225.6 | 6 | 9537.6 | 542.7 | 1 | 542.7 | 248.0 | 0 | – |
| 15% s in 2y olds, 1m lag | 95710.2 | 9 | 10634.5 | 66370.6 | 9 | 7374.5 | 937.1 | 2 | 468.6 | 249.6 | 1 | 249.6 |
| 15% s in 2y olds, 3m lag | 106664.39 | 8 | 13333.05 | 66652.94 | 9 | 7405.88 | 957.5 | 2 | 478.7 | 204 | 1 | 204 |
| 15% s in 2y olds, 6m lag | 106761.08 | 7 | 15251.58 | 64655.63 | 8 | 8081.95 | 730.5 | 2 | 365.3 | 182.58 | 1 | 182.58 |
