# Supplementary material for: Impact on Epidemic Measles of Vaccination Campaigns Triggered by Disease Outbreaks or Serosurveys: A Modeling Study
Source: PLoS Med. 2016 Oct 11;13(10):e1002144. doi: 10.1371/journal.pmed.1002144 (PMC5058560; doi:10.1371/journal.pmed.1002144)
Supplement: S3 Table — (DOCX) [file pmed.1002144.s007.docx]

|  | **Yemen-like** | | | **Niger-like** | | | **Nepal-like** | | | **Zambia-like** | | |
| --- | --- | --- | --- | --- | --- | --- | --- | --- | --- | --- | --- | --- |
|  | **cases**  **averted** | **TCUPS** | **c. averted/**  **TCUP** | **cases**  **averted** | **TCUPS** | **c. averted/**  **TCUP** | **cases**  **averted** | **TCUPS** | **c. averted/**  **TCUP** | **cases**  **averted** | **TCUPS** | **c. averted/**  **TCUP** |
| **10% of unvaccinated covered** | | | | | | | | | | | | |
| 10 cases, 1m lag | 13518.3 | 2 | 6759.2 | 9095.1 | 2 | 4547.6 | 491.5 | 1 | 491.5 | 548.6 | 1 | 548.6 |
| 10 cases, 3m lag | 14316.1 | 2 | 7158.0 | 8992.6 | 2 | 4496.3 | 502.4 | 1 | 502.4 | 475.6 | 1 | 475.6 |
| 10 cases, 6m lag | 12008.5 | 2 | 6004.3 | 6295.4 | 2 | 3147.7 | 330.9 | 1 | 330.9 | 277.3 | 1 | 277.3 |
| 25 cases, 1m lag | 15807.7 | 2 | 7903.8 | 9125.2 | 2 | 4562.6 | 410.6 | 1 | 410.6 | 378.0 | 0 | – |
| 25 cases, 3m lag | 15170.1 | 2 | 7585.1 | 9175.2 | 2 | 4587.6 | 335.1 | 1 | 335.1 | 351.7 | 0 | – |
| 25 cases, 6m lag | 12870.7 | 2 | 6435.3 | 6526.9 | 2 | 3263.5 | 367.7 | 1 | 367.7 | 263.6 | 0 | – |
| 10% s in 2-5y olds, 1m lag | 48807.7 | 10 | 4880.8 | 27979.1 | 10 | 2797.9 | 818.3 | 6 | 136.4 | 605.8 | 4 | 151.4 |
| 10% s in 2-5y olds, 3m lag | 46313.1 | 10 | 4631.3 | 27592.4 | 10 | 2759.2 | 850.1 | 6 | 141.7 | 534.4 | 4 | 133.6 |
| 10% s in 2-5y olds, 6m lag | 44324.1 | 10 | 4432.4 | 27255.8 | 10 | 2725.6 | 838.0 | 6 | 139.7 | 564.8 | 4 | 141.2 |
| 15% s in 2-5y olds, 1m lag | 33375.5 | 8 | 4171.9 | 20911.3 | 8 | 2613.9 | 279.6 | 1 | 279.6 | 146.1 | 0 | – |
| 15% s in 2-5y olds, 3m lag | 33182.7 | 7 | 4740.4 | 21249.3 | 8 | 2656.2 | 144.3 | 1 | 144.3 | 59.7 | 0 | – |
| 15% s in 2-5y olds, 6m lag | 35463.6 | 7 | 5066.2 | 20392.5 | 8 | 2549.1 | 248.4 | 1 | 248.4 | 171.4 | 0 | – |
| 15% s in 2y olds, 1m lag | 52130.8 | 12 | 4344.2 | 30597.9 | 11 | 2781.6 | 479.9 | 2 | 240.0 | 243.8 | 1 | 243.8 |
| 15% s in 2y olds, 3m lag | 52214.7 | 12 | 4351.2 | 30513.2 | 11 | 2773.9 | 514.9 | 2 | 257.5 | 277.4 | 1 | 277.4 |
| 15% s in 2y olds, 6m lag | 51357.1 | 11 | 4668.8 | 31189.4 | 11 | 2835.4 | 417.5 | 2 | 208.8 | 259.4 | 1 | 259.4 |
| **20% of unvaccinated covered** | | | | | | | | | | | | |
| 10 cases, 1m lag | 30905.6 | 2 | 15452.8 | 19791.4 | 2 | 9895.7 | 742.6 | 1 | 742.6 | 673.9 | 1 | 673.9 |
| 10 cases, 3m lag | 27611.9 | 2 | 13806.0 | 18205.7 | 2 | 9102.8 | 710.2 | 1 | 710.2 | 597.5 | 1 | 597.5 |
| 10 cases, 6m lag | 22982.0 | 2 | 11491.0 | 14196.8 | 2 | 7098.4 | 542.8 | 1 | 542.8 | 461.8 | 1 | 461.8 |
| 25 cases, 1m lag | 30519.2 | 2 | 15259.6 | 19715.8 | 2 | 9857.9 | 652.7 | 1 | 652.7 | 550.5 | 0 | – |
| 25 cases, 3m lag | 28523.6 | 2 | 14261.8 | 18351.0 | 2 | 9175.5 | 580.8 | 1 | 580.8 | 458.1 | 0 | – |
| 25 cases, 6m lag | 22801.0 | 2 | 11400.5 | 13284.6 | 2 | 6642.3 | 473.1 | 1 | 473.1 | 431.9 | 0 | – |
| 10% s in 2-5y olds, 1m lag | 84058.9 | 10 | 8405.9 | 53090.2 | 10 | 5309.0 | 1091.6 | 5 | 218.3 | 728.4 | 4 | 182.1 |
| 10% s in 2-5y olds, 3m lag | 83339.6 | 10 | 8334.0 | 52464.5 | 10 | 5246.5 | 1090.3 | 5 | 218.1 | 741.9 | 4 | 185.5 |
| 10% s in 2-5y olds, 6m lag | 79699.7 | 9 | 8855.5 | 49517.2 | 10 | 4951.7 | 1056.2 | 5 | 211.2 | 722.8 | 4 | 180.7 |
| 15% s in 2-5y olds, 1m lag | 66811.6 | 7 | 9544.5 | 41629.2 | 8 | 5203.7 | 332.7 | 1 | 332.7 | 47.9 | 0 | – |
| 15% s in 2-5y olds, 3m lag | 67753.5 | 7 | 9679.1 | 40909.8 | 8 | 5113.7 | 413.6 | 1 | 413.6 | 106.4 | 0 | – |
| 15% s in 2-5y olds, 6m lag | 59733.8 | 7 | 8533.4 | 37289.7 | 7 | 5327.1 | 314.4 | 1 | 314.4 | 115.8 | 0 | – |
| 15% s in 2y olds, 1m lag | 90188.0 | 11 | 8198.9 | 57592.6 | 12 | 4799.4 | 794.5 | 2 | 397.2 | 315.7 | 1 | 315.7 |
| 15% s in 2y olds, 3m lag | 88775.4 | 11 | 8070.5 | 55939.5 | 12 | 4661.6 | 698.4 | 2 | 349.2 | 325.0 | 1 | 325.0 |
| 15% s in 2y olds, 6m lag | 85937.0 | 11 | 7812.5 | 53779.3 | 11 | 4889.0 | 713.6 | 2 | 356.8 | 291.7 | 1 | 291.7 |
| **40% of unvaccinated covered** | | | | | | | | | | | | |
| 10 cases, 1m lag | 54213.9 | 2 | 27106.9 | 35822.2 | 2 | 17911.1 | 1067.9 | 1 | 1067.9 | 865.1 | 1 | 865.1 |
| 10 cases, 3m lag | 51521.0 | 2 | 25760.5 | 34596.9 | 2 | 17298.4 | 969.9 | 1 | 969.9 | 778.3 | 1 | 778.3 |
| 10 cases, 6m lag | 46018.1 | 2 | 23009.1 | 27932.2 | 2 | 13966.1 | 798.2 | 1 | 798.2 | 669.8 | 1 | 669.8 |
| 25 cases, 1m lag | 54917.8 | 2 | 27458.9 | 38053.9 | 2 | 19026.9 | 884.5 | 1 | 884.5 | 752.3 | 0 | – |
| 25 cases, 3m lag | 52601.9 | 2 | 26301.0 | 33828.6 | 2 | 16914.3 | 769.6 | 1 | 769.6 | 617.5 | 0 | – |
| 25 cases, 6m lag | 43617.2 | 2 | 21808.6 | 25071.3 | 2 | 12535.6 | 571.7 | 1 | 571.7 | 525.1 | 0 | – |
| 10% s in 2-5y olds, 1m lag | 95604.2 | 8 | 11950.5 | 65901.2 | 9 | 7322.4 | 1297.8 | 4 | 324.5 | 911.6 | 3 | 303.9 |
| 10% s in 2-5y olds, 3m lag | 95467.3 | 8 | 11933.4 | 65764.6 | 9 | 7307.2 | 1290.0 | 4 | 322.5 | 889.0 | 3 | 296.3 |
| 10% s in 2-5y olds, 6m lag | 95152.0 | 8 | 11894.0 | 65125.6 | 8 | 8140.7 | 1252.4 | 4 | 313.1 | 876.6 | 3 | 292.2 |
| 15% s in 2-5y olds, 1m lag | 90541.6 | 6 | 15090.3 | 60560.7 | 6 | 10093.5 | 598.1 | 1 | 598.1 | 308.1 | 0 | – |
| 15% s in 2-5y olds, 3m lag | 89488.1 | 5 | 17897.6 | 59566.4 | 6 | 9927.7 | 577.5 | 1 | 577.5 | 93.1 | 0 | – |
| 15% s in 2-5y olds, 6m lag | 85442.5 | 5 | 17088.5 | 56487.7 | 6 | 9414.6 | 522.0 | 1 | 522.0 | 223.4 | 0 | – |
| 15% s in 2y olds, 1m lag | 95532.1 | 9 | 10614.7 | 65769.5 | 9 | 7307.7 | 994.0 | 2 | 497.0 | 379.6 | 1 | 379.6 |
| 15% s in 2y olds, 3m lag | 95458.2 | 8 | 11932.3 | 65719.1 | 9 | 7302.1 | 993.5 | 2 | 496.7 | 523.06 | 1 | 523.06 |
| 15% s in 2y olds, 6m lag | 95050.9 | 8 | 11881.4 | 65139.3 | 8 | 8142.4 | 892.0 | 2 | 446.0 | 510.63 | 1 | 510.63 |
