# Supplementary material for: Impact on Epidemic Measles of Vaccination Campaigns Triggered by Disease Outbreaks or Serosurveys: A Modeling Study
Source: PLoS Med. 2016 Oct 11;13(10):e1002144. doi: 10.1371/journal.pmed.1002144 (PMC5058560; doi:10.1371/journal.pmed.1002144)
Supplement: S4 Table — Columns are as follows: mean total number of cases averted after 15 y across 200 simulations, mean total number of TCs and cases averted per TC for vaccination scenarios with the stated trigger, 20% coverage, and a 3-molag between trigger and vaccination. (DOCX) [file pmed.1002144.s008.docx]

|  | cases averted  (95% CI) | TCS  (95% CI) | cases averted per TC  (95% CI) |
| --- | --- | --- | --- |
| Yemen-like (73% routine coverage, 38 birth per 1,000) | | | |
| *10 cases* | 48,599 (45736, 51461) | 2.08 (2.02, 2.14) | 23,365 (21812, 24918) |
| *25 cases* | 47,551 (44749, 50354) | 1.99 (1.93, 2.05) | 23,895 (22285, 25505) |
| *10% s in 2-5y olds* | 93,972 (91669, 96276) | 9.90 (9.86, 9.94) | 9,492 (9256, 9728) |
| *15% s in 2-5y olds* | 88,293 (85907, 90678) | 7.46 (7.41, 7.51) | 11,835 (11506, 12165) |
| *15% s in 2y olds* | 94,217 (91914, 96520) | 11.26 (11.22, 11.29) | 8,371 (8164, 8578) |
| Niger-like (71% routine coverage, 48 birth per 1,000) | | | |
| *10 cases* | 26,930 (25127, 28733) | 2.27 (2.20, 2.34) | 11,863 (10993, 12733) |
| *25 cases* | 29,164 (27330, 30998) | 2.00 (1.94, 2.07) | 14,546 (13519, 15573) |
| *10% s in 2-5y olds* | 63,372 (61877, 64867) | 10.22 (10.18, 10.27) | 6,198 (6049, 6346) |
| *15% s in 2-5y olds* | 58,096 (56506, 59685) | 8.13 (8.09, 8.18) | 7,141 (6942, 7341) |
| *15% s in 2y olds* | 64,786 (63302, 66269) | 11.70 (11.67, 11.72) | 5,540 (5412, 5667) |
| Nepal-like (86% routine coverage, 24 birth per 1,000) | | | |
| *10 cases* | 999 (892, 1106) | 0.98 (0.94, 1.03) | 1,014 (894, 1134) |
| *25 cases* | 692 (579, 806) | 0.78 (0.74, 0.82) | 888 (735, 1040) |
| *10% s in 2-5y olds* | 1,234 (1127, 1340) | 5.06 (5.01, 5.11) | 244 (223, 265) |
| *15% s in 2-5y olds* | 465 (337, 594) | 1.15 (1.11, 1.19) | 405 (292, 517) |
| *15% s in 2y olds* | 899 (782, 1016) | 2.12 (2.07, 2.17) | 424 (368, 480) |
| Zambia-like (91%routine coverage, 46 birth per 1,000) | | | |
| *10 cases* | 510 (432, 589) | 0.88 (0.84, 0.92) | 580 (486, 674) |
| *25 cases* | 295 (211, 378) | 0.64 (0.61, 0.68) | 457 (325, 589) |
| *10% s in 2-5y olds* | 604 (521, 686) | 3.75 (3.70, 3.81) | 161 (139, 183) |
| *15% s in 2-5y olds* | 86 (-15, 188) | 0.36 (0.34, 0.39) | 237 (-41, 515) |
| *15% s in 2y olds* | 217 (123, 311) | 0.76 (0.72, 0.81) | 283 (160, 407) |
