# Supplementary material for: Impact on Epidemic Measles of Vaccination Campaigns Triggered by Disease Outbreaks or Serosurveys: A Modeling Study
Source: PLoS Med. 2016 Oct 11;13(10):e1002144. doi: 10.1371/journal.pmed.1002144 (PMC5058560; doi:10.1371/journal.pmed.1002144)
Supplement: S1 Text — (DOCX) [file pmed.1002144.s009.docx]

**Supplementary Methods to “Performance of Triggered Campaigns in Minimizing the Impact of Epidemic Measles in the Face of Uncertainty: a Modeling Study”**

Justin Lessler, C. Jessica E. Metcalf, Felicity T. Cutts, Bryan T. Grenfell

***Model structure***

To explore the impact of different triggered vaccination strategies for measles, we use a discrete-time model that incorporates both epidemic and demographic transitions, building on theory introduced in (1, 2); and used in (3, 4). We structure the population into age classes (1, 2, …., *z*; where *z* is the total number of age strata, here taken as *z*=83 with monthly age strata up to age 5 years, yearly age strata until age 20 years, and 5 year age strata thereafter up to age 60 years), and epidemiological classes (‘maternally immune’ M, ‘susceptible’ S, ‘infected’ I, ‘recovered’ R, and ‘vaccinated’ V, taken to indicate the effectively vaccinated, and only applied to susceptible individuals), and frame the joint processes of aging and infection as a matrix of demographic and epidemiological transitions.

Initially ignoring demographic transitions (survival and aging), within each age class *a* transitions between epidemiological categories occur according to:

$\mathbf{A}_{a,t}=\left( \begin{matrix} 1-d_{a} & 0 & 0 & 0 & 0 \\ d_{a} & 1-\varphi_{a}(\mathbf{n}\left( t \right))(1-v_{a}) & 0 & 0 & 0 \\ 0 & \varphi_{a}(\mathbf{n}\left( t \right))(1-v_{a}) & 0 & 0 & 0 \\ 0 & 0 & 1 & 1 & 0 \\ 0 & v_{a} & 0 & 0 & 1 \end{matrix} \right)$ (1)

The five rows and columns represent the M, S, I, R, and V categories, respectively, and the matrix contents capture transitions between them, where the time-step is taken as the approximate generation time of measles (5). In the transition matrix *d_a_* is the probability of losing maternal immunity, *φ_a_* is the probability of becoming infected, and *v_a_* is the probability of being effectively vaccinated. The infection probability *φ* (also called the force of infection, FOI) is a function of **n**(*t*), a vector describing the population at time *t*

$\mathbf{n}(t)=(M_{1,t},S_{1,t},I_{1,t},R_{1,t},V_{1,t},M_{2,t},\ldots,V_{z,t})^{\text{T}}$ (2)

according to

$\phi_{a}(\mathbf{n}(t))=1-\exp[-\sum_{j} \beta_{a,j,t}I_{j,t}^{\gamma}/\sum\mathbf{n}(t)]$ (3)

where *z* is the total number of age classes, *β_a,j,t_* is the rate of transmission between individuals in age classes *a* and *j*, referred to as the Who-Acquires-Infection-From-Whom or WAIFW matrix, and *γ* captures heterogeneities in mixing not directly modeled (5, 6) and the effects of discretization of the underlying continuous time process (7). Here we fix *γ* at 0.97, reflecting values obtained for measles in England and Wales (5). Discrete-time models that do not incorporate this exponent (i.e., *γ=*1) result in dynamics that are unrealistically unstable and prone to frequent extinction. Total population size appears as a denominator of number of infected individuals in each age class to reflect the fact that measles transmission scales in a frequency dependent manner (8).

Seasonality in transmission often plays an important role in the dynamics of childhood infections (9, 10). In the absence of detailed data, we chose to model seasonal fluctuations as a trigonometric function (*e.g.*, (9)), i.e., transmission to individuals in age strata *a*, from individuals in age strata *j* at time *t* are defined by $\beta_{a,j,t}=\beta_{1,a,j}(1+\beta_{2}\cos(2\pi t))$ where *β*_1,_*_a,j_* is mean transmission from individuals in age strata *j* to age strata *a*, and *β*_2_ is a parameter controlling the magnitude of seasonal fluctuations.

The full transition matrix **A**(**n**(*t*)) required to project the entire population forwards via aging, mortality and infection dynamics is defined by:

$\mathbf{A}(\mathbf{n}(t))=\left(\begin{matrix} s_{1}(1-u_{1})\mathbf{A}_{1} & 0 & 0 & ... & 0 \\ s_{1}u_{1}\mathbf{A}_{1} & s_{2}(1-u_{2})\mathbf{A}_{2} & 0 & ... & 0 \\ 0 & s_{2}u_{2}\mathbf{A}_{2} & s_{3}(1-u_{3})\mathbf{A}_{3} & ... & 0 \\ 0 & 0 & s_{3}u_{3}\mathbf{A}_{3} & ... & 0 \\ ... & ... & ... & ... & 0 \\ 0 & 0 & 0 & ... & .s_{z}\mathbf{A}_{z} \end{matrix}\right)$ (4)

where *s_a_* is the probability that an individual in age class *a* survives to the next time step, *u_a_* is the rate of aging out of age class *a,* and **A**_1_, **A**_2_, etc., are defined in equation (1), time-subscript dropped for convenience. The dynamics of the population as a whole can be projected forward according to the density dependent matrix model:

$\mathbf{n}(t+1)=\mathbf{A}(\mathbf{n}(t))\mathbf{n}(t)+\mathbf{B}_{t}$ (5)

where **B**(*t*) is a vector representing the number of births at time *t*

$\mathbf{B}_{\boldsymbol{t}}=(B_{t},0,0,\ldots, 0)^{T}$. (6)

Initial conditions were taken as values corresponding to the quasi-stationary distribution of the stochastic model for each set of parameters (obtained by iteration).

With this framework, we can calculate the basic reproductive ratio, *R*_0_ (the number of cases that would result from the introduction of a single infected individual into a completely susceptible population), as the dominant eigenvalue of the next generation matrix taken at the disease free equilibrium (1, 11, 12); and use this to calibrate *R*_0_ at a scale appropriate to measles (e.g., *R*_0_=20).

***Extensions to a stochastic framework***

We can extend the model to be stochastic and to include an immigration rate according to,

$\mathbf{n}(t+1)=S[\mathbf{A}(\mathbf{n}(t)),\mathbf{n}(t)]+\mathbf{B}_{t}+\mathbf{M}_{t}$ (7)

where in the stochastic setting, S[**A**(**n**(*t*)), **n**(*t*)] is a vector resulting from the sum of stochastic draws from multinomial distributions defined according to each column of the matrix **A** and the number of individuals in each category, **n**(*t*); **B*_t_*** is a vector with zeros corresponding to all but the first class, which in the stochastic setting is taken as a draw from a Poisson distribution around the time-varying mean birth rate, and **M*_t_*** is a vector with zeros corresponding to all but the infected classes, and a draw from a random Poisson distribution with mean *ι* for each of the infected classes (that is, we make the simplifying assumption that the immigration rate is the same for all age classes). Large values of *ι* correspond to high mobility and low coverage of adjacent locations; small values of *ι* correspond to low mobility and effective vaccination in adjacent locations.

***Parametrisation of demographic and epidemiological dynamics***

Given the absence of detailed data on measles dynamics for settings of interest, we combined estimates of known demographic rates for focal country contexts (e.g., birth rates per 1000 per year from http://data.worldbank.org/indicator/SP.DYN.CBRT.IN/countries; and mortality from the United Nations population division, <http://esa.un.org/wpp/Excel-Data/mortality.htm>) with model specification of the epidemiology designed to capture broad features of the biology of measles.

Specifically, the baseline demographic rates for each country context setting were used to define the stationary population age structure; and the total number of individuals from the capital city of the focal country setting was then distributed across the susceptible age classes accordingly. We also re-allocated appropriate numbers of individuals into the maternally immune category according to the probability of losing maternal immunity for individuals in the a^th^ age strata, *d_a_*, which was parametrized as an exponential decay with parameter 0.45 based on an average waning time between 3 and 9 months (13). We introduced an infected individual into the population and projected the population forward following equation 7 for 20 years to run out the transient dynamics. We then rescaled the population to the initial population size and introduced vaccination (see below) according to the scenario being explored.

Age assortative mixing is thought to be an important component of the dynamics of childhood infections like measles (14); with the key characteristic being high contact amongst younger individuals. Accordingly, we implemented age specific contact patterns (or a WAIFW matrix, as described above) following those characterized from diary studies in Europe (15). Although studies in other contexts have suggested some deviations from this basic pattern, the major features of the contact network appear to be conserved (16). The chosen WAIFW matrix was rescaled to reflect R_0_=20, likely to reflect measles dynamics in the contexts of interest (17).

Since there was no a priori reason to assume that our focal contexts reflected one country in this dataset more than another, we based our analysis on the full set of contacts recorded, aggregating according to our chosen age bins. We summed the resulting matrix with a transposed version of itself to ensure symmetry in contacts; and then smoothed the resulting surface using a 2D Kernel density estimator from the KernSmooth package in R.

***Parameterization of vaccination***

The probability of effective vaccination for individuals in the a^th^ age strata, v_a_ was obtained by multiplying probability of access to routine vaccination based on estimates from Zambia (18) with estimates of vaccine efficacy (19) (maximum is 0.97). Additionally, the pattern of access to routine vaccination was scaled to reflect desired coverage based on WUENIC estimates (20) for the focal country-context. Vaccination campaigns were implemented as brief (two week) interventions, that deliver vaccine to the proportion of susceptibles in their target age range following their coverage definition.

**References**

1. Klepac P & Caswell H (2010) The stage-structured epidemic: linking disease and demography with a multi-state matrix approach. *Theoretical Ecology* 4:301-319.

2. Klepac P*, et al.* (2009) Stage structured transmission of phocine distemper virus in the Dutch 2002 outbreak. *Proceedings of the Royal Society, Series B* 276:2469-2476

3. Metcalf CJE*, et al.* (2012) Structured models of infectious disease: inference with discrete data. *Theoretical Population Biology* 82 (4):275-282.

4. Metcalf CJE, Lessler J, Klepac P, Cutts FT, & Grenfell BT (2012) Minimum levels of coverage needed for rubella vaccination: impact of local demography, seasonality and population heterogeneity. *Epidemiology and Infection* 16:1-12.

5. Bjørnstad ON, Finkenstadt B, & Grenfell BT (2002) Endemic and epidemic dynamics of measles: Estimating epidemiological scaling with a time series SIR model. *Ecological Monographs* 72:169-184.

6. Finkenstadt B & Grenfell BT (2000) Time series modelling of childhood diseases: a dynamical systems approach. *Journal of the Royal Statistical Society, Series C* 49:187-205.

7. Glass K, Xia Y, & Grenfell BT (2003) Interpreting time-series analyses for continuous-time biological models-measles as a case study. *Journal of Theoretical Biology* 223:19-25.

8. Metcalf CJE, Munayco CV, Chowell G, Grenfell BT, & Bjørnstad ON (2011) Rubella meta-population dynamics and importance of spatial coupling to the risk of Congenital Rubella Syndrome in Peru. *Journal of the Royal Society Interface* 8:369-376.

9. Schenzle D (1984) Estimation of the basic reproduction number for infectious diseases from age-stratified serological survey data. *IMA Journal of Mathematics Applied in Medicine and Biology* 1:161-191.

10. Ferrari MJ*, et al.* (2008) The dynamics of measles in sub-Saharan Africa. *Nature* 451:679-684.

11. Diekmann O, Hesterbeek JAP, & Metz JAJ (1990) On the definition and the computation of the basic reproduction ratio R0 in models for infectious diseases in heterogeneous populations. . *Journal of Mathematical Biology* 28:365–382.

12. Allen L & van den Driessche P (2008) The basic reproduction number in some discrete-time epidemic models. *Journal of Difference Equations and Applications* 14:1127-1147.

13. Nicoara C, Zach K, Trachsel D, Germann D, & Matter L (1999) Decay of passively acquired maternal antibodies against measles, mumps and rubella viruses. *Clinical and Diagnostic Laboratory Immunology* 6:868-871.

14. Edmunds WJ, Gay NJ, Kretzschmar M, & Wachmann H (2000) The pre-vaccination epidemiology of measles, mumps and rubella in Europe: implications for modelling studies. *Epidemiology and Infection* 125:635–650.

15. Mossong J*, et al.* (2008) Social Contacts and Mixing Patterns Relevant to the Spread of Infectious Diseases. *PloS Medicine* 5:e74.

16. Read JM*, et al.* (2014) Social mixing patterns in rural and urban areas of southern China. *Proceedings of the Royal Society B: Biological Sciences* 281(1785):20140268.

17. Anderson RM & May RM (1991) *Infectious diseases of humans* (Oxford University Press, Oxford, OX2 6PD).

18. Lessler J, Lowther SA, Moss WJ, & Cummings DAT (2010) Maintaining high rates of measles immunization in Zambia. *Epidemiology and Infection* 5:1-11

19. Boulianne N*, et al.* (1995) Measles, mumps, and rubella antibodies in children 5-6 years after immunization: effect of vaccine type and age at vaccination. *Vaccine* 13:1611-1616.

20. WHO / UNICEF (WHO-UNICEF estimates of MCV coverage. ed <http://apps.who.int/immunization_monitoring/en/globalsummary/timeseries/tswucoveragemcv.htm> AtJ.
